# Financial losses for Dutch stakeholders during the 2013 aflatoxin incident in Maize in Europe

**DOI:** 10.1007/s12550-021-00429-9

**Published:** 2021-03-30

**Authors:** M. Focker, H. J. van der Fels-Klerx, A. G. J. M. Oude Lansink

**Affiliations:** 1 Business Economics, Wageningen University, Hollandseweg 1, 6708 WB Wageningen, The Netherlands; 2 Wageningen Food Safety Research (WFSR), Wageningen University and Research, Akkermaalsbos 2, 6708 WB Wageningen, The Netherlands

**Keywords:** Food incident, Food safety, Mycotoxins, Costs, Economics, Monte Carlo simulation

## Abstract

Early 2013, high concentrations of aflatoxin M_1_ were found in the bulk milk of a few dairy farms in the Netherlands. These high concentrations were caused by aflatoxin B_1_ contaminated maize from Eastern Europe that was processed into compound feed, which was fed to dairy cows. Since the contamination was discovered in the downstream stages of the supply chain, multiple countries and parties were involved and recalls of the feed were necessary, resulting into financial losses. The aim of this study was to estimate the direct short-term financial losses related to the 2013 aflatoxin incident for the maize traders, the feed industry, and the dairy sector in the Netherlands. First, the sequence of events of the incident was retrieved. Then, a Monte Carlo simulation model was built to combine the scarce and uncertain data to estimate the direct financial losses for each stakeholder. The estimated total direct financial losses of this incident were estimated to be between 12 and 25 million euros. The largest share, about 60%, of the total losses was endured by the maize traders. About 39% of the total losses were for the feed industry, and less than 1% of the total losses were for the dairy sector. The financial losses estimated in this study should be interpreted cautiously due to limitations associated with the quality of the data used. Furthermore, this incident led to indirect long-term financial effects, identified but not estimated in this study.

## Introduction

Aflatoxins are a worldwide issue for human and animal health as well as the economy. Although aflatoxins are more likely to be a problem in tropical and sub-tropical areas, they can also cause severe problems in other areas. For instance, the annual losses due to aflatoxins in US corn have been estimated to be about USD 163 million (Wu 2006). Aflatoxins have not been a major food safety issue in Europe in the past. However, high levels of aflatoxins have been observed in Southern and Eastern Europe in recent years. Due to climate change, aflatoxins are expected to become more and more an issue in Europe (van der Fels-Klerx, 2019). One of the most important aflatoxins is aflatoxin B_1_ (AFB_1_), which is frequently found in cereals, such as maize, and in nuts. Human exposure, via food intake, to AFB_1_ for a longer period of time, can lead to health complications such as immunotoxicity, hepatotoxicity, and teratogenicity (Eaton and Gallagher 1994; Kumar et al. 2017). Furthermore, when cows are fed with feed contaminated with AFB_1_, the toxin is metabolised in the cow’s body and excreted in the milk as the metabolite aflatoxin M_1_ (AFM_1_) (Hsieh et al. 1985; van der Fels-Klerx and Camenzuli 2016). In most countries, maximum (legal) limits are set for aflatoxins as a group and/or for AFB_1_ alone in food and feed products and/or for AFM_1_ in milk. In the European Union (EU), different limits are set for aflatoxins for different types of products. For example, in food, the legal limit for aflatoxins for all cereals and all products derived from cereals, including processed cereal products for AFB_1_, is 2 µg/kg. The legal limit for AFM_1_ in milk is 0.05 µg/kg (EU 2006). The legal limit for AFB_1_ in most feed products is 20 µg/kg; however, the limit for compound feed for dairy cattle is 5 µg/kg (EU 2002). European legal limits for aflatoxin are summarized in Table 1. Many feed companies in the Netherland use a lower limit for maize used in compound feed for dairy cows, being 2.5 µg/kg (SecureFeed, 2020).

**Table 1:** Legal limits for aflatoxins implemented in the European Union

Product	AFB_1_ (µg/kg)	AFM_1_ (µg/kg)	Reference
Human food			
Raw milk, heat-treated milk and milk for the manufacture of milk-based products		0.050	Commission Regulation (EC) No 1881/2006
Infant formulae and follow-on formulae, including infant milk and follow-on milk		0.025	
All cereals and all products derived from cereals including processed cereal products, with the exception of maize and rice subjected to sorting or other physical treatment before human consumption or use as ingredient in foodstuffs, processed cereal-based foods and baby foods for infants and young children, and dietary foods for special medical purposes intended specifically for infants	2.0		
Animal feed			
Feed materials with the exception of groundnut, copra, palm-kernel, cotton seed babassu, maize and products derived from the processing thereof	50.0		Directive 2002/32/EC
Feed materials from groundnut, copra, palm-kernel, cotton seed babassu, maize and products derived from the processing thereof	20.0		
Complete feedingstuffs for cattle, sheep and goats with the exception of dairy cattle, calves and lambs	50.0		
Complete feedingstuffs for dairy cattle	5.0		
Complete feedingstuffs for calves and lambs	10.0		

In February and March 2013, as part of regular monitoring, AFM_1_ was found to be present in the milk tanks of dairy farms in the Netherlands and in Germany. The source of this contamination turned out to be maize-based compound feed, fed to the dairy cows. Feed producers had (unintentionally) used maize that was contaminated with aflatoxins as ingredient in their compound feed production. During the maize growing season of 2012, high aflatoxin concentrations were observed in Southern and Eastern Europe (Popovic et al. 2017). Between July 2012 and July 2013, 17 RASFF alerts were published notifying AFB_1_ concentrations above the EU legal limit in maize intended to be used as feed ingredient (RASFF 2013). The contaminated maize originated from the Republic of Serbia, Bulgaria, Romania, Hungary, Ukraine, Spain, Italy, Greece, and Poland. Aflatoxins can already be present in the cultivation stage of the crops and, in case of improper conditions, can continue to be produced by their responsible fungi, notably *Aspergillus flavus*, during transport and storage. During compound feed formulation, the aflatoxin concentration usually will decrease since maize is just one of the ingredients used in the compound feed formulation and it is mixed with other ingredients. However, in case of high concentrations in the maize ingredient, the compound feed can be (highly) contaminated.

Even though the 2013 aflatoxin incident affected only four dairy farms in the Netherlands, this incident largely impacted the food safety control system of animal feed production in the Netherlands, up to today, which can be considered a severe consequence of the incident. The objective of this study was first of all to estimate the short-term direct financial impact for the Netherlands. Second, since the contamination was discovered in a relatively late stage of in the supply chain, at the level of the dairy farms, the objective was to estimate which stakeholders of the food supply chain (the maize traders, the feed producers or the dairy industry) suffered most losses. To the best of our knowledge, the estimated financial losses of any aflatoxin related incident, including the 2013 aflatoxin incident in Europe, has not been described before except for the country specific aspects of Serbia by Popovic et al (2017). Climate change may lead to an increased probability of the presence of aflatoxins (van der Fels-Klerx et al. 2019). Having knowledge of the potential financial impact of non-detected aflatoxin-contaminated feed, as well as the stakeholders most heavily impacted by these types of incidents, could help to decide upon prioritizing prevention and control at the different stages of the supply chain.

## Materials and methods

### Study demarcation

**Fig. 1:**
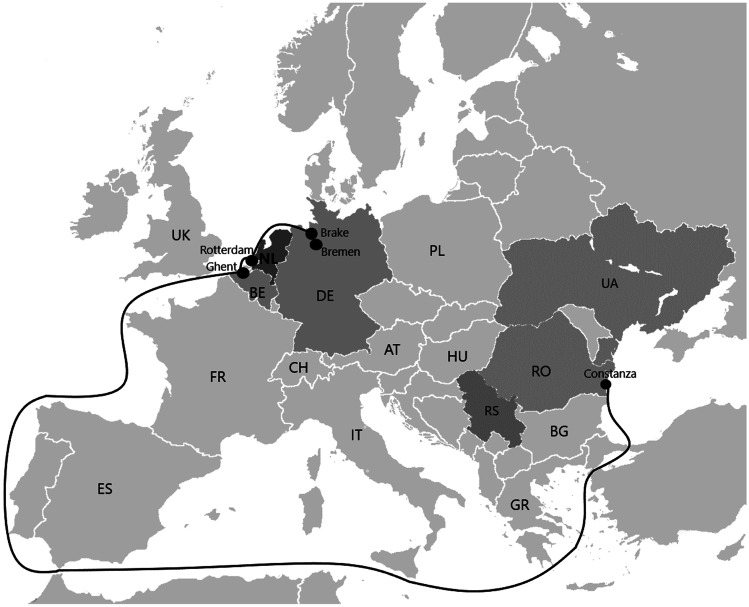
Map of the presumed route of the aflatoxin contaminated maize in 2012: from Eastern Europe, in particular from the Republic of Serbia, to the harbours of Rotterdam in the Netherlands, Ghent in Belgium, and Brake/Bremen in Germany. Abbreviations: *AT*, Austria; *BE*, Belgium; *BG*, Bulgaria; *CH*, Switzerland; *DE*, Germany; *ES*, Spain; *FR*, France; *IT*, Italy; *HU*, Hungary; *NL*, the Netherlands; *PL*, Poland; *RO*, Romania; *RS*, Republic of Serbia; *UA*, Ukraine; *UK*, United Kingdom

The origin of the contaminated maize was Eastern Europe and mainly the Republic of Serbia. The maize was then transported by ship to the Netherlands, Germany, or Belgium where the maize was stored or directly transported to compound feed companies for further processing (Fig. 1). The feed produced was transported to dairy farms where it was fed to dairy cows. The three main stakeholders of this incident involved from the Netherlands were included in this study; they were the maize traders, the feed producers, and the dairy farms using the feed. We considered a stakeholder of this incident, to be a group that is affected by the decisions of another group. Other stakeholders were not included in this study; amongst others, they include farmers growing the maize and the pig, poultry, and cattle farmers, excluding dairy farmers, using the contaminated feed and the governmental bodies. Farmers growing the maize were not included in this study since they were not located in the Netherlands and were therefore not within the focus of the study, even though, during the growing season of 2012, high aflatoxin concentrations were found in Eastern Europe and, consequently, the farmers as well as the dairy sector in this area did suffer from the contamination. This has been described previously (Popovic et al. 2017). Costs borne by governmental bodies were not included in the estimation of financial losses. Even though many staff from feed control and regulatory bodies were involved, we assumed these bodies set aside an annual budget for outbreak control. Furthermore, we assumed that the pig, poultry, and cattle farms, excluding dairy cattle farms, did not suffer from any significant losses since the feed for these animals did not exceed the EU legal limit and carryover of AFB_1_ from the feed to meat and eggs is very low (BfR 2013b).

## Information and data collection

Data were obtained from the literature and through experts’ interviews. First, we searched for scientific articles or reviews about the 2013 aflatoxin incident in the Netherlands using several databases: Scopus, Web of Science, PubMed, Agris, and Science Direct. The keywords used were “Aflatoxin” AND “milk” AND “2013.” Second, we searched for news items on the Internet for farmers and the general public and statements and reports written by authorities. The search was performed using Google NL. The keywords, in Dutch, “Aflatoxine” AND “melk” AND “2013” were used. Third, we searched for RASFF notifications of batches of maize intended to be used as feed materials that exceeded the legal limit for feed, as notified in the RASFF portal in the period 8 January 2012 to 7 January 2013. The product category was set to feed materials, the hazard category to mycotoxins, and the risk decision to serious (RASFF, 2013).

In order to complement the literature search and/or confirm the data found, five individual in-depth expert interviews were held. All interviewees were employed in the Netherlands. Two interviewees were working at a trading company, one interviewee was working at the Dutch Food and Consumer Products Safety, one interviewee was working at a Dutch feed producers’ association, and one interviewee was the owner of a feed producing company in the Netherlands. These experts were interviewed about quantities of contaminated maize imported, quantities of contaminated maize processed, quantities of dairy cow feed produced, and the quantity of feed recalled. They were also asked about the prices of maize and feed (per tonne), the costs for extra testing for AFB_1_ presence, recalling feed, and destroying maize and/or feed. A pre-defined questionnaire was used as the basis for the interviews.

## Financial impact model

This study focused on the direct short-term financial impact for three stakeholders in the Netherlands. Dairy milk exceeding the legal limit for AFM_1_ did not enter the consumer market; therefore, consumers as well as milk market prices were not affected. Furthermore, only a percentage of the maize incorporated in the feed was contaminated; supply shortages for both feed and milk did not occur, and therefore, the feed and milk prices did not rise. Since the contamination was discovered before the milk entered the market, the time effect was not considered in the analysis. Since the General Food Law states that any food business operator should only place safe food on the market all stakeholders were liable, the government did not compensate the maize traders, feed producers, or the farmers for their financial losses. The farmers were compensated for the testing of the milk and the feed by the feed producers.

A stochastic Monte Carlo model was developed in R-3.6.1. The model consisted of three stages in the supply chain: the import of the maize performed by the maize traders, the processing of the maize by the feed producers, and the production of milk at the dairy farms. The model results included the financial impact per stakeholder as well as the total financial impact. We chose a stochastic model, with 10,000 iterations, to include the uncertainty of the several input parameters: the number of tonnes of contaminated maize imported, the value of imported maize destined for feed, the percentage of maize used for feed, the percentage of maize used for dairy cattle feed, the cost-price to produce compound feed, the percentage of feed recalled, the costs to recall feed, the costs to destroy contaminated feed, the costs to test feed for AFB_1_, the costs to compensate dairy farmers, the number of cows per dairy farm, and the selling price for milk. The following input variables were considered to be deterministic variables: the value of maize intended for use as biogas, the number of dairy farms exceeding the legal AFM_1_ level, and the number of days a farm was blocked in case contaminated milk was found. A Monte Carlo simulation provides the entire possible range of outcomes, with their probabilities, depending on the distribution and correlation of values of the model input parameters.

The rest of this section describes the equations used in the model. The financial losses for the traders were based on the quantity of contaminated maize imported, not processed into feed but sold as biogas instead:

1 $$\begin{aligned}&\mathrm{Cost}\;\mathrm{maize}\;\mathrm{traders}\hspace{0.17em}\\& \quad=\hspace{0.17em}0.3\times import\times(100-q\_feed)\times(c\_import-c\_biogas)\end{aligned}$$

where *import* was the quantity of contaminated maize imported in tonnes, *q_feed* the percentage maize that was processed into feed, *c_import* the value of one tonne of imported maize destined for feed, and *c_biogas* the value of one ton of imported maize destined for biogas. Three ships containing contaminated maize were assumed to be partly processed in the Netherlands. It was assumed that the maize from these ships was distributed between the Netherlands, Belgium, and Germany proportionally to the animal feed production in these three countries. The Netherlands was producing 30% of the animal feed, Belgium 7%, and Germany 64% in 2013 (FEFAC, 2018). We therefore assumed that 30% of the incoming contaminated maize was processed in the Netherlands.

In order to estimate the financial losses for the feed industry, information was needed on the number of tonnes dairy of cattle feed produced using the imported contaminated maize. This was estimated with Eq. (2).

2 $$dairy\_feed\_cont\hspace{0.17em}=\hspace{0.17em}0.3 \times import \times q\_feed \times q\_dairy \times (1/q\_maize)$$

In Eq. (2), *dairy_feed_cont* was the quantity, in tonnes, of dairy cattle feed produced with the contaminated maize, *import* the quantity of contaminated maize imported in tonnes, *q_feed* the percentage maize that was processed into feed, *q_dairy* the percentage of feed produced destined for dairy cattle, and *q_maize* the percentage of maize incorporated into the compound feed.

Then, information was needed on the volume of feed (tonnes) that was recalled. This was estimated with Eq. (3):

3 $$t\_recall\hspace{0.17em}=\hspace{0.17em}(q\_recall/100) \times dairy\_feed\_cont$$

where *t_recall* was the number of tonnes of feed recalled, *q_recall* the percentage of feed delivered to the dairy farmers that was recalled, and *dairy_feed_cont* the quantity, in tonnes, of feed produced, calculated with Eq. (2).

The total recall costs, including the destruction and the replacement of the feed, were estimated by the following equation:

4 $$tc\_recall\hspace{0.17em}=\hspace{0.17em}t\_recall \times (c\_recall\hspace{0.17em}+\hspace{0.17em}c\_destr\hspace{0.17em}+\hspace{0.17em}c\_feed)$$

where *tc_recall* was the total costs to recall, destroy, and replace the feed; *t_recall* the quantity of feed recalled, in tonnes; *c_recall* the costs, per tonne, to recall the feed; *c_destr* the costs, per tonne, to destroy the feed, and *c_feed*, the costs, per tonne, to replace the feed.

Next, the costs for *extra* testing of contaminated Eastern European unprocessed maize stored at the feed producers’ premises or dairy cow’s compound feed produced from contaminated Eastern European maize were estimated using the following:

5 $$tc\_test\_feed\hspace{0.17em}=\hspace{0.17em}c\_test\_feed \times dairy\_feed\_cont$$

where *tc_test_feed* was the total costs for testing the raw maize and feed, *c_test_feed* the average testing costs per tonne of feed and *dairy_feed_cont* the quantity, in tonnes, of (contaminated) dairy cattle feed produced.

The costs to compensate the dairy farmers who received contaminated feed were estimated by the following equation:

6 $$tc\_comp\hspace{0.17em}=\hspace{0.17em}c\_comp \times dairy\_feed\_cont$$

where *tc_comp* was the total costs for the feed industry to compensate the dairy farmers who received the feed, *c_comp* the compensation costs per tonne of feed, and *dairy_feed_cont* the quantity of feed produced from contaminated maize.

The total direct costs for the feed industry were estimated with Eq. (7).

7 $$\mathrm{Costs}\;\mathrm{feed}\;\mathrm{industry}\hspace{0.17em}=\hspace{0.17em}tc\_recall\hspace{0.17em}+\hspace{0.17em}tc\_test\_feed\hspace{0.17em}+\hspace{0.17em}tc\_comp$$

In Eq. (7), *tc_recall* was the total costs to recall the feed, *tc_test_feed* the total costs for extra testing, and *tc_comp* the total costs to compensate the dairy farmers.

To estimate the financial losses for the dairy industry, the assumption was made that if the milk produced at a particular farm was above the legal limit, the farm could not sell any milk for some time after the discovery of the contamination. Farms receiving contaminated feed but not having the milk exceeding the legal limit did not suffer any major costs. The costs for the milk testing were most of the times declared to the feed industry, and added to the financial losses for the feed industry. Furthermore, no milk was lost. The test results were available the same day. The financial losses for the dairy sector were computed using Eq. (8).

8 $$\begin{aligned}&\mathrm{Cost}\;\mathrm{dairy}\;\mathrm{sector}\hspace{0.17em}\\& \quad=\hspace{0.17em}nb\_farms\_cont\times nb\_days\times nb\_cow\times l\_milk\times c\_milk\end{aligned}$$

where *nb_farms_cont* was the number of farms at which AFM_1_ was found above the legal limit, *nb_days* the number of days after the discovery that the dairy farms were unable to sell their milk, *nb_cows* the number of cows per farm, *l_milk* the litres milk produced per cow per day, and *c_milk* the selling price of 1 l of milk for a farmer.

## Results

Three scientific articles related to the 2013 aflatoxin incident were found. De Rijk et al. (2015) used one batch of maize involved in the 2013 aflatoxin incident to investigate the efficiency of the EU sampling procedures (de Rijk et al. 2015). Van der Fels-Klerx and Camenzuli (2016) used the concentrations observed during the 2013 aflatoxin incident to model the possible AFM_1_ concentrations in milk (van der Fels-Klerx and Camenzuli 2016). Popovic et al. (2017) described the financial losses of the 2013 aflatoxin incident for the Serbian dairy sector. None of these scientific papers described the events of the 2013 aflatoxin incident in detail, and neither of them estimated the financial losses for the Netherlands. Relatively, many news items, reports, and announcements were available on the web. Based on this grey literature, we were able to describe the events, as presented in the section hereafter. After the description of events, a list of RASSF notifications in which the Netherlands was involved is presented. The last section of the results presents the financial losses of this incident.

### Description of the events based on grey literature

In the Netherlands, in February 2013, AFM_1_ was found to be present in the milk tanks of two dairy farms (Boerenbusiness 2013b; NRC 2013). Six weeks later, AFM_1_ was again found to be present in the milk of two other Dutch farms (Veeteelt 2013). The milk did, however, not exceed the EU legal limit (Boerenbusiness 2013e).

The high levels of AFM_1_ found in the Netherlands were the result of contaminated compound feed that was fed to the dairy cows. This compound feed included maize contaminated with AFB_1_ coming from Eastern Europe (Boerenbusiness 2013b, 2013e). Three contaminated maize batches were identified: one ship containing maize from the Republic of Serbia which entered Germany in the port of Brake, one ship with maize from the Republic of Serbia which entered the Netherlands in the port of Rotterdam, and one ship with maize from Romania which entered Belgium in the port of Ghent (EVMI 2013).

#### Batch 1

Before February 2013, in Germany, 45,000 tonnes of AFB_1_-contaminated maize from the Republic of Serbia were imported in the port of Brake in North Germany, via the port of Constanza in Romania. After detection of high concentrations of AFB_1_ in the maize batch, about 10,000 tons were blocked in Brake, and 25,000 tonnes was stored in a warehouse in Bremen and were blocked there. The remaining 10,000 tonnes of maize were already delivered and processed into feed in Germany and in the Netherlands (BfR 2013a; Boerderij 2013; EVMI 2013; Niedersächsisches Ministerium für Ernährung Landwirtschaft und Verbraucherschutz 2013; Zeit 2013). The compound feed produced by Dutch feed producers, using the contaminated maize, was mostly delivered and fed to pigs in the Netherlands (EVMI 2013).

#### Batch 2

On February 20th, a 45,000-t contaminated maize batch from the Republic of Serbia entered the Netherlands via the harbour of Rotterdam. About 1000 tonnes was blocked in Rotterdam. About 1000 tonnes was delivered to feed producers in Germany on 21 February. On March 6th, about 1200 tonnes was delivered to Germany but could be traced and blocked before being processed (Boerenbusiness 2013f). Another source states that 35,000 tonnes was stored in Bremen, Germany (NOS 2013a), and about 10,000 tonnes was processed by feed producers and delivered to about 3600 farms in Germany and in the Netherlands (Blik op Nieuws 2013; NRC 2013). However, an estimate of the Dutch news agency NOS pointed at 6500 farms (NOS 2013b). The feed produced was delivered to 87 dairy farms, which needed to be tested for the presence of AFM_1_ before they could sell their milk again. After 3 days, the testing results showed no AFM_1_ concentrations above the EU legal limit in the milk tested, and the affected farms could sell their milk again (Niedersächsisches Ministerium für Ernährung Landwirtschaft und Verbraucherschutz 2013).

#### Batch 3

One 53,000-t contaminated maize batch from Romania was delivered to Belgium in the port of Ghent. The batch was then transported to the Netherlands. The largest part of the batch was blocked before further processing. Part of the batch was processed into feed for pigs, poultry and, though to a lower extent, for cattle. The AFB_1_ concentration in the compound feed did not exceed the legal limit. Furthermore, the milk samples collected from dairy farms using the feed did not show high levels of AFM_1_ (EVMI 2013; VRT 2013). It was stated that the feed was not used in Belgium. All samples of raw materials, feed products, and milk analysed in Belgium were compliant to the EU limits for AFM_1_ (FASFC 2013).

#### Measures taken

Dutch feed producers arranged recalls of their feed. The recalls took approximately 2 weeks. First, compound feed with high inclusion rates of maize from the Republic of Serbia, Romania, and Hungary was recalled and, next, feed with lower percentages of maize from Eastern Europe was recalled as well (Boerenbusiness 2013a, 2013d). TRUST FEED, the umbrella organisation of compound feed producers in the Netherlands, states that the produced feed did not exceed the EU legal limit for AFB_1_, the feed contained on average 1 µg/kg AFM_1_, and the recalls were a preventive measure (TRUST FEED 2013). The contaminated maize was not used for feed, nor for biogas, and was either destroyed or sold to the USA (Boerenbusiness 2013c).

The Dutch organisation TRUST FEED gave advice not to include maize from the Republic of Serbia, Romania, and Hungary into dairy cattle feed. Furthermore, TRUST FEED stated that the feed producers intensified the monitoring program for aflatoxins of incoming maize batches. In addition to sampling when unloading the ship, extra samples were collected when the batches arrived at the processing plants (Trust Feed 2013).

### RASFF notifications

Three contaminated sea ships were reported in the news items mentioned before; however, 17 RASFF notifications of aflatoxins in maize destined as feed were made between 8 January 2012 and 7 January 2013 (RASFF, 2013). If only the notifications involving the Netherlands were considered, eight notifications were left. One batch was returned to the consignor; two batches were used for other purposes than feed or food; two batches were officially blocked; for two batches, the recipients had to be informed; and for one batch, the decision was unknown. The list and details of the notifications are presented in Table 2.

**Table 2:** List of RASFF notifications notifying maize batches, destined as feed, contaminated with aflatoxins distributed to the Netherlands

Date	Origin	Distribution	Type of check	AFB_1_ (µg/kg)	decision
01/March/2013	RO	NL, DE, via BE	Company own check	57.6–71.3	Informing recipients
01/March/2013	RS	DE, NL, US, via RO	Official control	204, 112, 38, 21	Official detention
04/March/2013	RS, RO, BG, PL	DE, UK, via NL	Company’s own check	37.1	Official detention
08/March/2013	RO, RS, BG	BE, FR, DE, NL	Company’s own check	1.9–158.5	/
13/March/2013	RO, BG	DE via NL	Company own check	22.4–26.7	Use for other purpose than food/feed
19/March/2013	HU	DE, NL, AT	Company own check	117.5–102.5	Return to consignor
29/March/2013	UA	BE, FR, DE, NL	Company’s own check	32.1	Informing authorities
16/March/2013	UA	BE, DE, via CH and NL	Company’s own check	35.4	Use for other purpose than food/feed

*AT* Austria, *BE* Belgium, *BG* Bulgaria, *CH* Switzerland, *DE* Germany, *FR* France, *HU* Hungary, *NL* the Netherlands, *PL* Poland, *RO* Romania, *RS* Republic of Serbia, *UA* Ukraine, *UK* United Kingdom, *US* United States

### Estimation of the financial losses

**Fig. 2:**
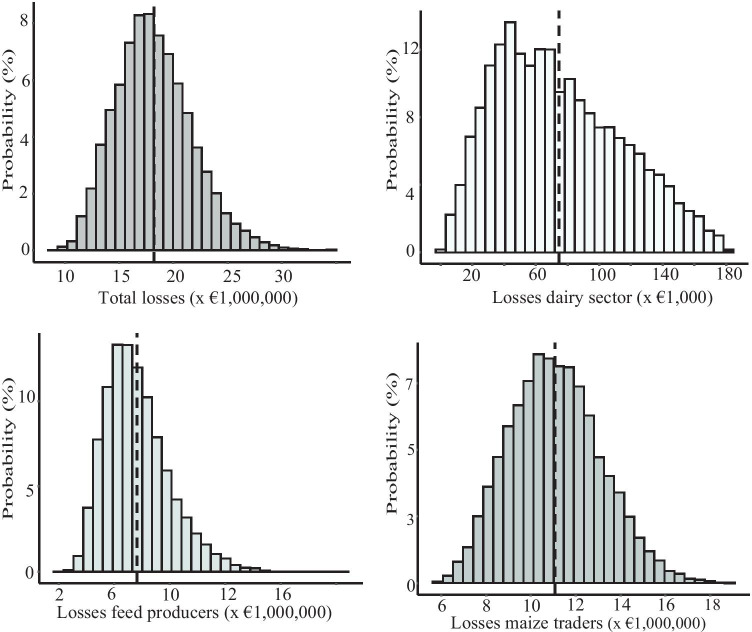
Estimated direct costs for the maize traders, the feed producers, and the dairy sector in the Netherlands, Belgium, and Germany due to the 2013 aflatoxin incident

Table 3 presents the collected data based on news items available on the web, RASFF notifications, scientific literature, some publicly available statistics, and expert interviews. The experts interviewed are or were working in the feed industry, trading companies, or the Netherlands food safety authority. In case only one data point was available, and this data point was uncertain, a relative standard deviation (RSD) of 25% around that data point was assumed. Based on these collected data, input values on the model parameters, as shown in Table 3, were determined. The estimation of the direct costs related to the 2013 aflatoxin incident, resulting from the Monte Carlo simulations, is presented in Fig. 2 and Table 4.

**Table 3:** Model variables and data collected to estimate the costs of the 2013 aflatoxin incident for different stakeholders in the Netherlands

Variable	Abbreviation	Unit	Estimations	Source	Estimation/distribution used in the model
Tonnes of contaminated maize imported from Eastern Europe in 2012	import	t	1) 143,000 2) 225,000 3) 300,000	1) 45,000 t + 45,000 t + 53,000 t 2) 3 ships each between 70,000 and 80,000 t ^(1)^ 3) 4 ships (RASFF, 2013), 75,000 t ^(1)^	Triangular (143,000; 300,000; 225,00)
Value maize from Eastern Europe in 2012	c_import	€/t	1) 220–260 2) 244–255 3) 279–255	1) FOB Black sea region (Potori and Józsa 2014), 20 €/t for maritime transport added ^(2)^ 2) Prices between Nov 2012 and Jan 2013 ^(3)^ 3) Prices between Aug and Dec 2012 (Agrimatie 2012)	Normal (251; 17)
Value maize intended for use as biogas	c_biogas	€/t	30	(Boerenbond 2014)	30
Percentage imported maize processed	q_feed	%	1) 22 2) 28	10,000 t (batch 1) + 10,000 t (batch 2), which is 22% (NVWA 2014) ^(5)^	Normal (0.25;0.063)
Percentage of total feed production used as dairy cattle feed	q_dairy_feed	%	22	The Netherlands, 2013 (FEFAC 2018)	0.22
Percentage of maize in dairy cattle feed	q_maize	%	1) 10–20 2) 10	(Devun et al. 2014; van der Fels-Klerx and Camenzuli 2016) ^(4)^	Uniform (0.10;0.20)
Cost price compound feed	c_feed	€/t	1) 240–300 2) 305	(Remmelink 2018) ^(4)^	Triangular (220; 279; 251)
Percentage feed recalled	q_recall	%	60	^(4) (5) (6)^	Normal (0.60; 0.15)
Recall costs	c_recall	€/t	58	^(4) (5) (6)^	Normal (58; 14.5)
Costs destruction feed	c_destr	€/t	5	^(4) (5) (6)^	Normal (5; 1.25)
Costs testing feed for AFB_1_	c_test_feed	€/t	5	^(4) (5) (6)^	Normal (5; 1.25)
Costs compensation dairy farms	c_comp	€/t	84	^(4) (5) (6)^	Normal (84; 21)
Number of dairy farms exceeding the legal AFM_1_ level	nb_farms_cont	n	4	( Veeteelt 2013)	6
Number of days a farm was blocked in case of contaminated milk	nb_days	n	14	^(2)^	14
Number of dairy cows per farm	nb_cows	n	Min: 1 Mean: 83 Max: 400	(CBS 2017; NZO 2016)	Triangular (1; 400; 83)
Litres of milk produced	l_milk	l/day	21	(The Daily Milk 2017) ^(5)^	21
Price per litre milk	c_milk	(€/l)	0.39	Average milk price in the Netherlands in 2013 (EU, 2017)	0.39

^(1)^Dutch Association Feed Companies, personal communication

^(2)^Own estimation

^(3)^Dutch trading company, personal communication

^(4)^Dutch Feed company, personal communication

^(5)^Calculation based on information available

^(6)^Assumption: RSD of 25%

**Table 4:** The estimated financial losses for the maize traders, the feed producers, the dairy sector, and the total financial losses related to the 2013 aflatoxin incident

	Mean	Median	5% percentile	95% percentile
Direct costs maize traders	11,044,800 €	10,929,800 €	7,892,400 €	14,568,800 €
Direct costs feed producers	7,047,400 €	6,510,900 €	2,678,400 €	2,678,400 €
Direct costs dairy sector	74,100 €	68,400 €	19,500 €	147,100 €
Direct total costs	18,166,400 €	17,796,100 €	12,508,400 €	25,159,000 €

Figure 1 and Table 4 show the distributions of the estimated direct financial losses for the maize traders, the feed producers, the dairy farms, and the total estimated financial losses. The largest percentage of the direct financial losses, about 60%, is for the maize traders, with an average estimated cost of about 11 million €, with the 5th percentile being 7.9 million € and the 95th percentile 14.5 million €. The direct financial losses for the feed producers are in the order of 7 million €, between 2.7 and 13.4 million €. The direct financial losses for the dairy sector are almost negligible with a mean of 74,100 €. The total financial losses are in the order of 18.1 million €, with the 5th percentile being 12.5 million € and the 95th percentile being 25.2 million €.

## Discussion

The probability of the concentration of AFB_1_ being above this limit is, in general, small because only a small percentage of maize is incorporated in the feed and the EU limit for AFB_1_ in feed is 20 µg/kg (EU 2002). Given that the EU limit for compound feed for dairy cows feed is 5 µg/kg (EU 2002), the probability of the AFB_1_ concentration exceeding this limit is much higher. Furthermore, AFB_1_ is metabolised in the cow’s body and is excreted as AFM_1_ in the milk. Since AFM_1_ is an unwanted toxic compound, contaminated maize can lead to both dairy cow feed exceeding the EU limit for AFB_1_ and dairy cows’ milk exceeding the EU limit for AFM_1_ (van der Fels-Klerx and Camenzuli 2016). In 2013, both dairy cow feed and the milk produced exceeded the EU limits for aflatoxins. Information was collected from various sources to estimate the financial losses of the 2013 aflatoxin-maize incident for the Dutch maize traders, feed producers, and dairy farms. Maize from several contaminated ships were imported from Eastern Europe into Germany, Belgium, and the Netherlands and were partly processed by Dutch feed producers.

The total financial losses for the Netherlands were estimated to be between 12 and 25 million € with the largest share (about 60%) for the maize traders. The incident was initially discovered in the milk of a handful of dairy farms in February 2013. However, it was quickly traced back to contaminated maize used in feed production. Due to extra testing of the incoming maize batches, the incident remained relatively small and mostly at the level of the maize traders. Since the AFB_1_ concentrations discovered in maize in several ship compartments were above the EU legal limit for maize intended for feed production, the maize had to be returned to the supplier, used for other purposes, or destroyed. This study showed this had led to high direct financial losses.

The financial losses for the feed industry, about 39% of the total financial losses, were mostly due to the maize which had already been processed before discovering the high AFM_1_ concentrations in the milk. About 22% of the maize coming from the three imported shipments was processed into feed for pigs, poultry, and cattle. Only a small percentage of this maize was used in compound feed for dairy cattle. The direct financial losses for the dairy sector, estimated at less than 1% of the total financial losses, were negligible compared to the total financial losses. However, for the individual farms having their milk exceeding the EU limit, the financial losses could be high: 2 weeks of closure of the farm being equivalent to roughly 3.8% of the yearly income.

In this study, we considered only the direct financial losses of the 2013 aflatoxin incident. We assumed that the indirect, short-term, financial losses, such as a lower export of dairy products and a lower domestic consumption of dairy products, were negligible for the Netherlands since no contaminated milk had reached the markets. From 2012 to 2013, the export of Dutch dairy products increased by 12% in general. In 2013, 60% was exported to five countries: Germany, Belgium, France, the UK, and Russia. In 2012, about 187 million of dairy products were exported to Russia. In 2013, this export increased to 300 million (Business Insider Nerderland 2014). From these numbers, we conclude that the export volume did not suffer from the 2013 aflatoxin incident. Since the milk sold on the market did not exceed the EU legal limit for AFM_1_, there were no expected risks for the consumers. In the Republic of Serbia, the situation was different: it was estimated that the loss for the dairy sector mounted to 96.2 million € (Popovic et al. 2017).

Farmers in Eastern Europe suffered immensely from the high aflatoxin concentrations in maize during the growing season of 2012. In the Republic of Serbia, the crisis lasted for 2 years and affected all dairy companies in the country. Popovic et al. (2017) estimated that the total direct and indirect economical losses of the Serbian farm-level dairy sector during the crisis mounted to 74.4–96.2 million € depending on the scenario. The Republic of Serbia has about 158,000 dairy farms and is a net exporter of dairy products. During the crisis, in the Republic of Serbia, the consumption of dairy products decreased by 11.4%, up to even 26.6% in the city Belgrade. Export of dairy products decreased as well, e.g. the export of liquid dairy products was halved. In February 2013, due to the extremely high levels of aflatoxins in Serbian maize, the Serbian Minister of Agriculture changed the legal limit for AFM_1_ in milk from 0.05 to 0.5 µg/kg, the legal limit applicable in the Republic of Serbia until 2011. Under the pressure of the crisis, the Minister resigned in June 2013. The next Serbian Minister of Agriculture set the legal limit back to 0.05 µg/kg, the same limit as the one used in the EU, starting in April 2014. However, this new regulation proved impossible to control and apply since maize from the summer of 2012 was still in use. A third Minister of Agriculture changed the Serbian legal limit to 0.25 µg/kg, before setting it back to the EU limit of 0.05 µg/kg in January 2015 (Popovic et al. 2017).

In this study, the losses were estimated with the assumption that maize traders were unaware of the contamination. However, at least for the ship entering Germany, several aspects point towards criminal energy. Serbian maize was imported by a large feed trader, who either did not control the maize or hid the high aflatoxin levels. German authorities prosecuted this feed trader but did not convict him. Losses for the maize traders could be overestimated in this paper due to the fact that no criminal intentions were assumed. If contaminated maize would have been intentionally bought at a discount price, costs would have been reduced, leading to reduced losses for the traders. Furthermore, we assumed that the contaminated raw maize was classified as unfit for animal or human consumption and sold as biogas instead and that the produced dairy cow feed was destroyed and not fed to other animal nor sold to other countries. However, at least a part of the raw maize and dairy cow feed exceeding the EU limits for aflatoxins was sold to the USA, where higher legal limits are in place. It is plausible that the maize was sold at a higher price to the USA than the price that would have been paid by biogas producers in Europe.

Furthermore, Wu (2008) estimated the average direct financial losses for an EU border rejection of a cereal batch because of mycotoxin contamination, to be between 8900 € and 13,400 € for extra costs, including sampling, testing, transportation, storage, labour costs, and reprocessing (Wu 2008). Additional costs can occur depending on the destination of the batch for, since it might still be suitable for feed instead of food. These administrative costs are not included in this study and could be added to the financial losses for the maize traders. The losses for the governmental bodies were not considered in this study since we assumed that these bodies have reserved budgets for possible incidents. The outbreak control costs made by the governmental bodies during the 2013 *Salmonella* Thompson incident in the Netherlands were estimated to be approximately 9% of the total losses (Suijkerbuijk et al. 2016). Therefore, we expect that including the losses for the governmental bodies would not change dramatically our estimation of the total losses for the Netherlands.

The Netherlands has suffered from a couple of other food incidents in the past years. Each incident led to very different losses for different stakeholders in the food chain. For example, the losses due to the *Salmonella Thompson* outbreak caused by smoked salmon in the Netherlands in 2012–2013 were estimated at 7.5 million €. These losses consisted of the cost-of-illness corrected for underestimation and the outbreak control costs made by the governmental bodies (Suijkerbuijk et al. 2016). Unlike the *Salmonella* incident, the 2013 aflatoxin incident did not lead to cost-of-illness in the Netherlands. Despite of this, the estimated financial losses were higher than the losses estimated for the *Salmonella* incident. The losses due to the fipronil incident in the Netherlands in 2017 were estimated to be between 65 and 75 million €, with more than 50% of the losses for the laying hen farmers (van Horne et al. 2017). The estimated losses for the 2013 aflatoxin incident were much lower than the losses estimated for the fipronil incident, also an incident without any illnesses but with much more Dutch companies involved.

The 2013 aflatoxin incident did have other indirect, long-term, consequences in the Netherlands, which are not considered in this study. Changes were made to the Dutch control program for aflatoxin in maize. The feed industry in the Netherlands has intensified their monitoring program for aflatoxins in maize after the 2013 aflatoxin incident. Since then, extra sample collection is required when unloading batches in the harbour, at the level of the ships transporting the maize to the feed producers. Furthermore, after the 2013 aflatoxin incident, countries exporting maize to the Netherlands are classified each year into low, medium and high risk countries for aflatoxin contamination. Each category of countries has its own monitoring intensity, which leads to extra costs for monitoring in the medium and high risk countries. In addition, since the 2013 aflatoxin incident, up until early 2019, compound feed producers test one batch of feed that contains maize as ingredient per week for the presence of aflatoxins; the frequency is now lowered to once a month (SecureFeed, 2020). Given that there are 40 plants producing feed for dairy cattle in the Netherlands and aflatoxin testing costs of a batch are between 300 and 1100 € when 20 or 100 samples are collected, respectively (Focker et al. 2019), this would lead to an extra 624,000 to 2,288,000 € per year for the feed industry, when one batch per week per location is tested.

The estimation of the financial losses of the 2013 aflatoxin incident was based on scarce data that had to be obtained from different sources and combined. In order to account for the uncertainty of the input data, a Monte Carlo simulation was performed. Since for some variables, we could collect only one data point, a relative standard deviation of 25% was added to this data point. However, uncertainty still remains and the results presented in this study are only an approximation of the financial losses for the three stakeholders in the Netherlands.

To conclude, the results of this study showed that imported maize with an aflatoxin concentration above the EU legal limit for feed lead to high direct financial losses, first of all for the maize traders, and also for the feed producers in case the maize has already been processed. Increasing the frequency of monitoring, in the upstream stages of the maize supply chain, could help avoid financial losses for stakeholders in the downstream stages of the maize supply chain, such as the feed producers and the dairy sector.

## Data Availability

All data are provided in the manuscript.

## Code availability

The code of the model used is available upon request.
